# Evolving Cell-Based and Cell-Free Clinical Strategies for Treating Severe Human Liver Diseases

**DOI:** 10.3390/cells9020386

**Published:** 2020-02-07

**Authors:** Viviana Cernigliaro, Rossella Peluso, Beatrice Zedda, Lorenzo Silengo, Emanuela Tolosano, Rinaldo Pellicano, Fiorella Altruda, Sharmila Fagoonee

**Affiliations:** 1 Department of Molecular Biotechnology and Health Sciences, University of Turin, Via Nizza 52, 10126 Turin, Italy; vivianacerni@hotmail.com (V.C.); rossellapeluso14@gmail.com (R.P.); beatrice.zedd@gmail.com (B.Z.); 2 Maria Pia Hospital, 10126 Turin, Italy; 3 Molecular Biotechnology Center, Departmet of Molecular Biotechnology and Health Sciences, University of Turin, Via Nizza 52, 10126 Turin, Italy; lorenzo.silengo@unito.it (L.S.); emanuela.tolosano@unito.it (E.T.); 4 Unit of Gastroenterology, Molinette Hospital, 10126 Turin, Italy; rinaldo_pellican@hotmail.com; 5 Institute of Biostructure and Bioimaging, National Research Council, Molecular Biotechnology Center, Via Nizza 52, 10126 Turin, Italy

**Keywords:** liver diseases, transplantation, cell therapy, extracellular vesicles, organoids, scaffolds, organ printing

## Abstract

Liver diseases represent a major global health issue, and currently, liver transplantation is the only viable alternative to reduce mortality rates in patients with end-stage liver diseases. However, scarcity of donor organs and risk of recidivism requiring a re-transplantation remain major obstacles. Hence, much hope has turned towards cell-based therapy. Hepatocyte-like cells obtained from embryonic stem cells or adult stem cells bearing multipotent or pluripotent characteristics, as well as cell-based systems, such as organoids, bio-artificial liver devices, bioscaffolds and organ printing are indeed promising. New approaches based on extracellular vesicles are also being investigated as cell substitutes. Extracellular vesicles, through the transfer of bioactive molecules, can modulate liver regeneration and restore hepatic function. This review provides an update on the current state-of-art cell-based and cell-free strategies as alternatives to liver transplantation for patients with end-stage liver diseases.

## 1. Introduction

Cirrhosis and hepatocellular carcinoma cause approximately 2 million deaths per year, placing liver disorders among the top 20 most common causes of death worldwide [1]. Chronic exposure to excessive and prolonged use of alcohol, viral infections, metabolic disorders, toxins, non-alcoholic fatty liver and cancer are among the common causes of liver cirrhosis [2]. Advanced cirrhosis is generally considered irreversible, unlike its preceding steps (hepatitis and fibrosis), even when the causal agent is removed [3]. Currently, liver transplantation (LT) is the only viable alternative to reduce cirrhosis-induced mortality rates [4]. Given the importance of LT, much progress has been made regarding surgical and conservation techniques. Surgical improvements have mainly focused on the phases of reconstruction and anastomoses. For instance, Carmody et al. recently compared biliary transposition to recipient biliary ductoplasty for biliary reconstruction, and showed that both techniques were useful in the case of significant bile duct size mismatch [5]. Regarding liver conservation, ex vivo normothermic machine perfusion of the organ safely and efficiently extends its conservation time until transplanted, hence allowing transport for longer distances. The physiological conditions of the organ (temperature, nutrients and oxygen) can thus be maintained outside the body and the risk of ischemic reperfusion injury prevented [6,7]. Moreover, Patrono et al. recently reported that hypothermic oxygenated machine perfusion reduced ischemia-reperfusion injury in liver grafts from brain-dead donors [8].

Scarcity of donor organs is the main limitation for LT. Thus, other surgical approaches have been studied, such as the use of “marginal” organs, and partial LT from living donors [9,10]. The marginal organs are obtained from donors even over the age of 60, and with hypernatremia and steatosis greater than 40% or with positive serology for hepatitis C (HCV) or B (HBV) virus as well [11]. However, with this strategy, there are limits related to post-transplantation survival which, to be bypassed, require a careful selection of donors. The partial transplantation, on the other hand, adopts the split technique, through which a liver is divided and transplanted to two patients (two adults or an adult and a child weighing less than 10 kg), hence permitting living-donor LT to be performed [10]. However, complications such as small for size syndrome or those of the biliary and vascular pathways, especially in recipients with high Model for End-Stage Liver Disease (MELD) scores, may ensue. Despite the fact that, in case of liver diseases caused by viral infections, new antiviral treatments have permitted significant advances, the risk of recidivisms that require a re-transplantation for other severe liver diseases remains a major obstacle [12,13,14]. Moreover, inflammatory responses and acute or chronic immune-mediated organ rejection, life-long requirement of immunosuppressive drugs, and incidence of postoperative infections following LT are still unresolved issues. Another hassle regards the finding of post-transplantation fibrosis upon evaluation of liver biopsies for histological changes in the long term [15]. Analysis of liver biopsies after 12 months or more post-LT in pediatric patients receiving liver allografts has revealed different degrees of inflammation and fibrosis, despite revealing normal liver function parameters [15,16]. Sinusoidal fibrosis and pericellular fibrosis are also commonly encountered in liver biopsy specimens following LT [17].

A possible alternative for LT may be xenotransplantation. To date, the only xenotransplant from pig donor to human has been performed in a 26-year-old patient with fulminant hepatitis, hepatic encephalopathy (HE) and coagulopathy [18]. Xenotransplantation led to an improvement in bile production, lactate clearance and stabilization of prothrombin times, decreased serum bilirubin levels, and transaminases. However, no neurological improvement was observed and the patient died 34 h after the xenotransplantation [18]. Other studies have been performed in non-human primates with genetically-engineered porcine livers and the recipient’s survival almost reached one month [19]. Thus, this strategy may be considered as a bridge therapy prior to LT in patients for whom no alternative is available. A potential limitation to this approach could be the transmission of porcine endogenous retroviruses. In the near future, following completion of more advanced preclinical studies, it will be possible to consider undertaking clinical trials [20,21,22].

It is to be noted that ethical concerns arise with liver transplantation, such as employing deceased donor organs, transplantation of HCV-infected donor livers into uninfected patients and their subsequent treatment with a direct-acting antiviral regimen, allocation of organs, and living donor transplantation [23,24]. Thus, alternative strategies are urgently required to overcome these problems related to LT. New resolute and lasting interventions need to be implemented to restore correct liver function. In recent years, cell-based and cell-free strategies as well as evolving technologies have shown promises as therapeutic alternatives in patients with end-stage liver diseases, when the liver’s regenerative capacity is impaired and endogenous liver stem cells can no longer cope with chronic insults. To this end, the present review aims at summarizing the current state of cell-based and cell-free alternatives to LT for patients with severe liver diseases.

## 2. Cell Therapy

### 2.1. Hepatocyte Transplantation

Hepatocytes, obtained from donor organs, can be transplanted without complex surgery into recipients for restoring hepatic function. These cells are isolated, using standardised perfusion techniques with collagenase, from human livers that are unsuitable for transplantation or from liver segments available after split transplantation [25]. Fresh hepatocytes can be delivered through intraportal, intrasplenic or intraperitoneal routes, or cryopreserved for use on demand. Usually 5–10% of the total hepatic mass must be substituted to obtain therapeutic benefits, and multiple infusions are often necessary.

The first hepatocyte transplantion in humans dates back to 1992 for the treatment of cirrhotic patients. However, the results of this first autologous transplantation were uncertain [26]. Since then, hepatocyte transplantation has been extended to other liver pathologies, including those induced by metabolic defects, such as urea cycle disorder and Crigler–Najjar syndrome. For instance, Fox et al. transplanted allogeneic hepatocytes into the liver of a 10-year old patient with Crigler–Najjar Syndrome type I, and observed clinically relevant long-term (up to 11 months) functioning of transplanted human hepatocytes conferring partial metabolic recovery [27]. The first European hepatocyte transplantation in adults was performed in a glycogen storage disease type 1a patient, and resulted in partial correction of metabolic abnormalities that lasted beyond 9 months [28]. Several transplantation schemes have been adopted with promising results (Table 1). Hepatocyte transplantation has also been performed in a case of fulminant hepatic failure induced by mushroom intoxication. A patient in hepatic coma following the ingestion of *Amanita phalloides,* and with very high values of International Normalized Ratio (INR) and Factor V, was infused with vital primary hepatocytes and with steroids and cyclosporine A as immunosuppressant over 30 h. Improvement in hepatic function ensued, and interestingly, signs of recurrence were absent, rendering it possible to suspend immunosuppression [29].

Importantly, hepatocyte transplantation can be used as bridging therapy awaiting organ transplantation (bridge to transplant) or for liver regeneration (bridge to recovery). Despite its advantages such as the lower invasiveness, repeatability, possibility of leaving the endogenous organ to promote self-regeneration, and individual autologous approach, hepatocyte transplantation still faces unmet challenges such as recovering enough viable cells from non-transplantable organs, in vitro culture and expansion without reduction in functionality, and cryopreservation without viability loss. Furthermore, post-transplantation problems include low engraftment of hepatocytes, and the need for immunosuppressive therapy due to the high antigenicity of hepatocytes [25]. Attempts to improve hepatocyte engraftment and repopulation in the recipient’s liver, thus giving a selective advantage to transplanted cells, for instance through partial hepatectomy, portal embolization or irradiation of the liver, are currently ongoing, and have been extensively reviewed in [30]. Moreover, the choice of donor organ for hepatocyte isolation is crucial. Importantly, the results of hepatocyte transplantation in 5 adult patients with acute liver failure and 4 pediatric ones with inborn metabolic disorders showed that this procedure is safe and feasible, as long as viable and metabolically functional human hepatocytes are employed [31,32]. Livers with more than 40% steatosis or from the elderly have lower hepatocyte yield, viability and survival after cryopreservation, and are therefore not recommended for hepatocyte isolation for transplantation [33].

### 2.2. Stem Cell Therapy

**Figure 1:**
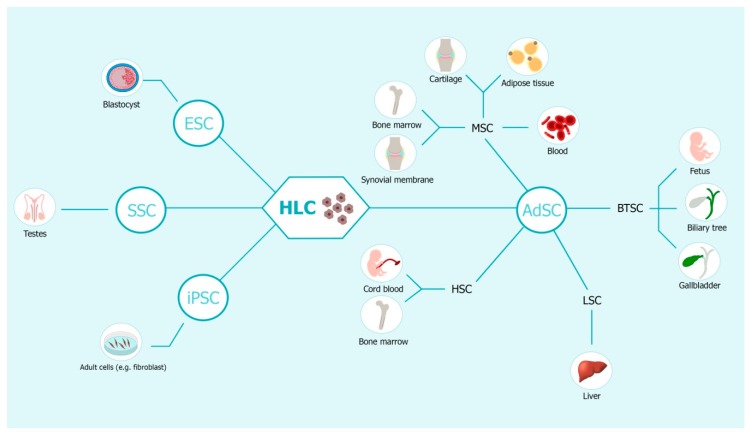
Sources of hepatic-like cells (HLCs) for stem cell therapy in liver disease. HLCs can be differentiated from embryonic stem cells (ESCs) derived from the inner cell mass of blastocysts, or from adult stem cells (AdSCs). The main types of AdSCs used for cell therapy are: mesenchymal stem/stromal cells (MSCs) isolated from blood, adipose tissue, cartilage, bone marrow and synovial membrane; hematopoietic stem cells (HSCs) found in the bone marrow and umbilical cord blood; biliary tree stem/progenitor cells (BTSCs) derived from the peribiliary glands of the adult and fetal human biliary tree or from the crypts of the gallbladder; endothelial progenitor cells (EPCs) taken from peripheral vessels and from bone marrow; liver stem cells (LSCs) localised in the liver. HLCs can be also obtained from induced pluripotent stem cells (iPSCs) obtained by reprogramming of adults cells by specific growth factors or spermatogonial stem cells (SSCs) derived from testis.

The limitations of hepatocyte transplantation have encouraged the search for other alternatives to LT. Stem cells have become the most promising candidates for liver cell replacement due to their expandability and differentiation potential. Stem cells derived from embryonic or adult tissues can be induced to differentiate into Hepatocyte-Like Cells (HLCs) under specific culture conditions (Figure 1) and show promises for the treatment of severe liver diseases. Adult Stem Cells (AdSCs) offer the possibility of autologous transplantation and of overcoming ethical constraints compared to Embryonic Stem Cells (ESCs), and include Mesenchymal Stem/Stromal Cells (MSCs), hematopoietic stem cells (HSCs), Endothelial Progenitor Cells (EPCs), Liver Stem Cells (LSCs), induced Pluripotent Stem Cells (iPSCs) and Spermatogonial Stem Cells (SSCs) (Figure 1). The use of these cells as well as other types of stem cells for liver therapy is discussed below.

#### 2.2.1. HSCs and EPCs in Liver Repair

**Figure 2:**
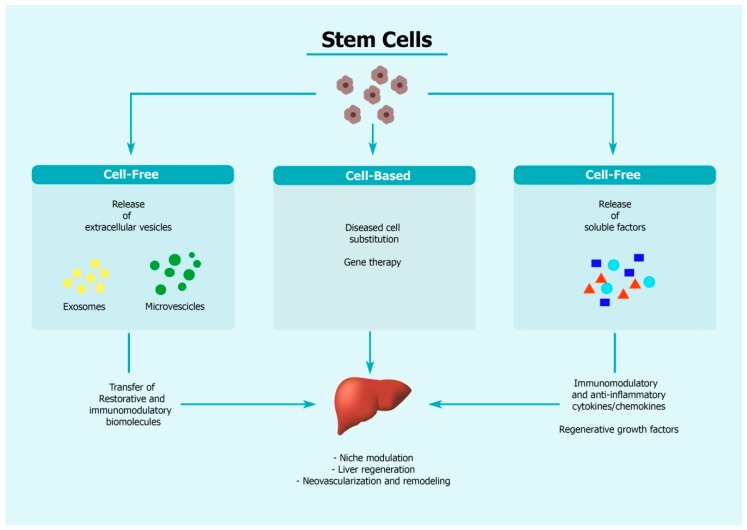
The mechanism of action of stem cells in the treatment of liver diseases. Stem cell injection may act in several ways in supporting liver repair. Functional stem cells may substitute diseased liver cells and at the same time provide the wild-type gene in case of genetic deficiencies, hence serving as a platform for gene therapy. Stem cells also release soluble factors such as growth factors and cytokines/chemokines to dampen liver injury. Extracellular vesicles (EVs) harbouring biomolecules with restorative properties are also produced by stem cells and participate in liver regenerative process.

HSCs originate in the embryonic liver, and successively migrate for definitive hematopoiesis to the bone marrow. They are highly plastic, showing differentiation into hematopoietic lineages as well as other non-hematopoietic lineages such as hepatic oval cells, hepatocytes, skeletal muscle cells, lung epithelial cells and cardiomyocytes [39]. Following liver damage, HSCs are mobilized in the peripheral circulation and are recruited to the site of injury [40]. HSCs may induce repair either through transdifferentiation into or fusion with hepatocytes or through the release of paracrine factors (Figure 2) [40]. Several clinical trials have been undertaken with HSCs for the treatment of liver cirrhosis; however, the outcome of the studies remains unclear [41].

#### 2.2.2. MSCs in Liver Repair

MSCs are considered one of the most effective multipotent cells capable of promoting transdifferentiation into hepatocytes, cell proliferation and neovascularization. MSCs, derived from different tissues, have been found to home to damaged liver and to contribute to its repair mainly through different mechanisms including their anti-inflammatory and immunomodulatory actions, (Figure 2) [42,43,44]. The mechanisms by which MSCs exert their therapeutic effects in models of liver cirrhosis are manifold as largely revealed from preclinical studies, and include activation of autophagy and downregulation of transforming growth factor (TGF)-β pathway [45], modulation of the key enzymes involved in glucose homeostasis [46], inhibition of activated stellate hepatic cells, decrease in collagen deposition, and increased remodelling of the extracellular matrix (ECM) [47]. However, MSCs scarcely engraft in the damaged area due to the inflammatory and toxic microenvironment. Strategies to improve MSC function and survival have been tested, and involve MSC priming approaches with inflammatory cytokines (for example, Tumor Necrosis Factor (TNF)α, Interferon (IFN)γ), hypoxic conditions, pharmacological drugs and chemical agents such as valproic acid, use of biomaterials (in spheroids) and different culture conditions (for example, addition of lipopolysaccharides) (extensively reviewed in [48]).

Several clinical trials have been carried out using MSCs, for instance, in patients suffering from HBV-related cirrhosis, in whom the regulation of Treg/Th17 cells was observed [49], and in patients with alcoholic cirrhosis who showed a decrease in TGF-β1, COL1A1 and α-smooth muscle actin levels [50]. Suk et al., in patients with alcoholic cirrhosis, confirmed a reduction of hepatic collagen deposition and an increase in both liver function and MELD score after MSC transplantation [51]. Improved liver function was also observed in two clinical trials involving, respectively, 26 and 60 patients with autoimmune liver cirrhosis and hepatolenticular degeneration [52,53]. Further research is needed to define more precisely the therapeutic window and the optimal cell dosage required to further the benefits, as well as to clarify current controversies regarding MSC transplantation in the management of patients with liver fibrosis [54].

#### 2.2.3. Liver Stem Cells

LSCs represent another potential candidate for cell transplantation (Table 2). Using different approaches, several groups have isolated liver cells with stem cell properties from the human liver. The most studied for their liver regenerative capacities hitherto are the liver MSC-like cells. These cells express markers of mesenchymal cells such as vimentin and α-smooth muscle actin, as well as those of hepatocytes including albumin and several subtypes of cytochrome P450 [55,56]. Some cells also express pluripotency markers such as Oct4 and nanog [57]. Their propensity to engraft and restore liver function has been demonstrated in preclinical studies involving animal models of severe liver diseases [58]. For instance, we have recently demonstrated that human liver MSC-like cells (human LSCs or HLSCs) are capable of restoring UGT1A1 enzyme activity in an immunocompromised mouse model of Crigler–Najjar Syndrome type I (CNSI) and of improving the phenotype [59]. The safety of liver MSCs has also been evaluated in a Phase I/II clinical trial in patients affected by urea cycle disorder and Crigler–Najjar syndrome (Table 2) [60]. The results are very encouraging. The authors showed that a low incidence rate of adverse events and a very low rate of serious adverse events occurred 1 month after cell infusion [60]. Human liver MSCs could also partially reinstate metabolic activity in these patients. Equally encouraging results were reported very recently with human LSCs (HLSCs). LSCs were injected in pediatric patients with inherited neonatal-onset hyperammonemia for clinical safety evaluation (Table 2) [61]. Importantly, patients were not subjected to treatment with immunosuppressive agents, due to the low immunogenicity of the cells infused. Cell injection did not induce any adverse events or intra-and extra-hepatic complications. Steady levels of ammonia were found in these patients, despite an increase in protein intake by approximately 30%, showing the capacity of these human LSCs to offer a bridge therapy untill the newborns are ready to undergo LT [61].

Other types of stem cells in the human liver include the hepatobiliary progenitor cells, known as “oval” cells in mice. These cells were recently identified using single-cell RNA sequencing technology, and showed a distinct gene expression profile compared to other liver parenchymal populations [62]. The bipotentiality of these cells was shown upon differentiation into TROP-2/CK9-positive (biliary cells) or albumin/HNF4α-positive (hepatocytes). These cells also are of great interest for liver regeneration and further studies will witness their utility in human liver regeneration.

#### 2.2.4. Adult Pluripotent Stem Cells and Transdifferentiated Cells

The pluripotent stem cells *par excellence* are the ESCs, which have paved the way to identifying and creating the next-generation of pluripotent stem cells. However, due to ethical constraints, human ESCs are not yet readily employed in the clinic. Research on hESCs is still ongoing. To this end, recently, clinical grade functional hepatocytes have been generated from human ESCs, and biosafety evaluation was performed in preclinical studies [63]. Whether these cells may be used in patients still needs to be addressed in terms of immunocompatibility and ethical limitations.

IPSCs have great potential in the field of liver regeneration. IPSCs, derived from the reprogramming of adult cells, share ESC characteristics and have an unlimited capacity for differentiation but are not subject to ethical concerns. HLCs derived from iPSCs (iHLCs) using different approaches have shown hepatocyte functionality in vitro and in preclinical models as well as potential for liver disease modelling and drug testing [64,65]. Several cell sources were employed in iHLCs generation, and the question regarding which source is the best for efficiently generating mature and transplantable hepatocytes capable of restoring liver function, still remains open. Recently, primary liver cells obtained through liver needle biopsy were also successfully reprogrammed into iPSCs and functional hepatocytes, but the latter had a distinct transcription profile with respect to the originating liver, suggesting that the tissue of origin does not impact much on the differentiation efficiency of iPSCs [66]. Despite the success in the generation of hepatocytes derived from iPSCs for transplantation, there is still a need to improve and solve the old challenges of engraftment and repopulation [67]. To date, no clinical trials with iPSC-derived-hepatocytes as a therapeutic alternative to LT have been carried out.

Interestingly, somatic cells obtained from simple biopsies can undergo lineage reprogramming to generate functional human HLCs. While a direct lineage reprogramming was initially used to generate hepatocytes by transduction, for instance, with a cocktail of factors including HNF4α, this approach resulted in functional cells that had to be expanded through SV40 large T antigen introduction, for example [68,69]. Recently, a two-step conversion process was used by passing through the generation of expandable human hepatic progenitor cells, followed by the induction of hepatocyte maturation [70]. This approach can be used to obtain sufficient functionally-competent hepatocytes for transplantation in patients.

Spermatogonial stem cells (SSCs) also show promise for liver regeneration. SSCs are derived from adult testes, and have the propensity to convert to pluripotent stem cells sharing features with ESCs in vitro. We and others have demonstrated that mouse SSCs can be efficiently induced to differentiate into functional HLCs in vitro, and that the transplanted HLCs engraft into mice livers [71,72,73,74,75]. The pluripotency characteristics of human SSCs are still being investigated. However, human SSCs also show high plasticity and were successfully used to generate functional HLCs in vitro. Chen et al. reported the direct transdifferentiation of human SSCs to bipotent hepatic stem cells expressing both hepatic and cholangiocyte markers, and then to mature and functional hepatocytes [76]. The potentiality of the SSCs for human liver regeneration requires further assessment in clinical studies.

#### 2.2.5. Current Limitations of Cell Therapy

Despite the panoply of beneficial effects, there are still unmet challenges regarding cell-based therapy. For instance, the time taken to produce GMP (Good Manufacturing Practice)-grade cells for clinical use is too long, which is worsened by regulatory challenges and financial burden. Cytogenetic abnormalities may result from long-term cell culture and passages, and rigorous controls are required before use in patients. Cell counting and cell viability evaluation are fundamental aspects in these studies. Moreover, the percentage of cells engrafting in the liver is still very low and the underlying mechanisms responsible for their beneficial effects are not completely understood [77]. Achieving enough cell engraftment in histologically normal livers capable of conferring therapeutic benefits, such as in the case of CNSI, remains untackled. Loss of functional properties of injected cells may also occur over time. Different cell types require different delivery routes, and the cell source as well as dose and number of injections need to be optimised preclinically based on the liver disease etiology in order to avoid toxicity. In addition, the clinical use of ESCs and iPSCs, albeit their differentiation capacity into HLCs, are hampered by the risk of teratoma formation from possible residual cells with pluripotent properties. Another major concern regarding stem cell-based therapy regards the possibility of liver fibrosis and hepatocellular carcinoma development over time [78]. All these concerns have solicited the search for alternative and improved strategies.

### 2.3. Recent Improvements in Clinical Cell-Based Strategies

#### 2.3.1. Encapsulation

To overcome some of the limitations of the use of cells as alternatives to LT, new methods have been devised. For instance, encapsulation of cells before transplantation provides controlled release of a wide range of drugs, cytokines, growth factors and hormones [22]. Cells are incorporated in polymerized, biocompatible and semi-permeable structures, called microspheres or microcapsules, which are composed of biologically active materials with adjustable permeability such as alginate [79]. The bidirectional diffusion of oxygen and metabolic products needed for cell survival and expansion, the control of the differentiation process towards a specific lineage, and the protection from host’s immune attack render this approach very attractive in the field of regenerative medicine [80].

Several cell types have been encapsulated for applications in different fields of tissue engineering, such as pancreas, myocardial, endoderm and bone tissue repair [81]. Human hepatocyte microbeads, generated in polymerized alginate, showed hepatocyte-specific function and lack of immunogenicity in vitro [82]. Moreover, transplantation of these microbeads intraperitoneally in rats provided metabolic support and rescued them from acute liver failure. Recently, iPSCs were differentiated in a 2D monolayer followed by 3D aggregation and further encapsulation in alginate capsules, resulting in enhanced hepatocyte phenotype or function compared to conventional culture conditions [83]. Furthermore, encapsulated human co-cultures were transplanted into immunocompetent mice without causing immune rejection for at least 24 days, showing their clinical potential [83].

Several aspects of the microbead systems need improvement, such as their relatively low physical strength as well as the capsule instability due to ionic bonds between calcium ions and alginate. The physiological exchange of calcium ions with sodium ions also causes osmotic swelling and destabilization of the microcapsules. To overcome these problems, a new combination of sodium alginate with polyethylene glycol (PEG) has been developed; this confers greater mechanical strength and stability [79]. However, additional strategies that reduce potential fibrotic reactions and improve vascularisation should be considered as a further clue for the applicability of the encapsulation strategy in the clinical setting.

#### 2.3.2. Bioartificial Liver Device

**Figure 3:**
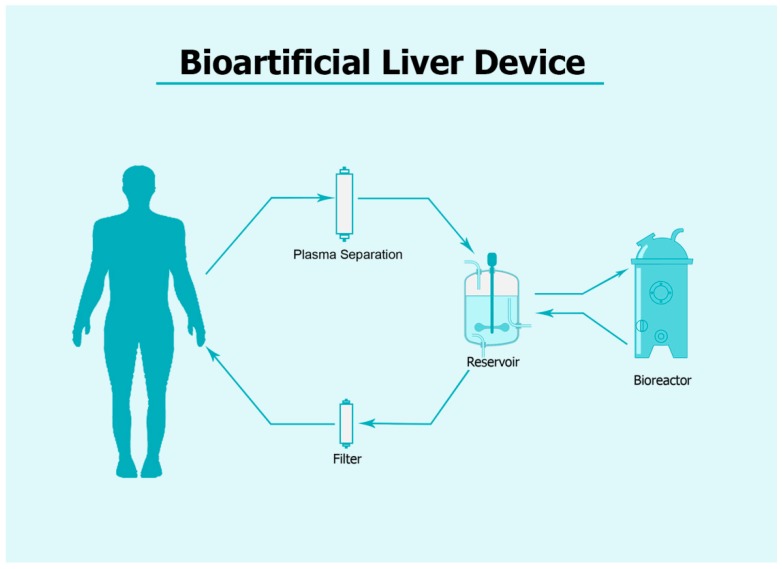
A Bioartificial Liver (BAL) system: Patient blood is taken from the venous circulation and separated from plasma, which flows into a reservoir through a pump system. The plasma then goes into a bioreactor inoculated with living cells and returns to the patient after filtration and rejoins the blood.

The increase in the number of patients awaiting LT and the inability of support systems to restore liver function have led to the advent of extracorporeal bioartificial liver (BAL) devices [2]. BAL devices are support systems for liver function, which perform detoxification and synthesis, for instance, and are connected to the patient’s venous circulation with the possibility of plasma separation (Figure 3). The latter flows through the bioreactor where liver cells have been seeded for metabolic exchange, and plasma is subsequently returned to the patient [84]. Based on their configuration, BAL devices are classified into systems based on hollow fibers, multi-layer membranes or a sponge/scaffold base, and floating/encapsulated. Hollow fiber devices are the most used in clinical studies. The ideal cellular source has not yet been identified. Primary human hepatocytes are useful for these systems but cannot be seeded in BAL devices for clinical studies due to their low availability and quality [85]. Only cells similar to highly functional hepatocytes derived from pluripotent stem cells showed potential [86]. These cells expressed hepatocyte markers, and demonstrated hepatic functions. IPSCs, which cannot yet be used in other applications due to their tumorigenic potential, are very useful in BAL systems as these cells would be isolated from the patient’s blood by multiple layers of filtering membranes. Thus, while iPSC-derived liver cells may not be ideal for cell transplantation, these cells are valid candidates for the BAL system [87].

One of the most studied BAL devices is the extracorporeal liver assist device (ELAD), which uses the human hepatoblastoma cell line HepG2 C3A in hollow fiber-based dialysis cartridges [87]. In this system, the cells grow inside the extracapillary space of a cartridge while the patient’s plasma flows inside the lumen of the hollow fibers. The latter, made with a semi-permeable membrane, allow the passage of the patient’s ultrafiltrate to C3A cells while allowing the exchange of toxins and nutrients [2]. Another BAL support system, HepatAssist, employs pig hepatocytes within an extracapillary compartment of a hollow fiber bioreactor [2]. Despite the wide availability of porcine hepatocytes, these cells raise some concerns in terms of xenotransplantation in humans, due to the possibility of xenozoonosis [88]. A solution is presented by the work of Sauer et al. that developed the extracorporeal hepatic modular support device (MELS) using primary human hepatocytes in a 3D framework of hollow fiber membranes [89].

The effectiveness of BAL systems has been investigated by numerous clinical trials [87]. The safety and efficacy of HepatAssist has been attested in the first prospective, randomized, controlled trial of an extracorporeal liver support system on patients with fulminant/subfulminant hepatic failure. Survival was significantly higher in the BAL group compared to the control group (73% versus 59%) [90]. ELAD was tested in a phase III trial, which recruited 203 patients with alcoholic hepatitis, of whom 96 were treated with ELAD and 107 with SMT. Comparison of the basic characteristics between the two groups did not reveal any significant difference. However, a regression analysis highlighted high levels of creatinine, but not of bilirubin. ELAD could potentially benefit young subjects with sufficient renal function and less severe coagulopathy [91]. In a trial involving 8 patients (2 with ALF, 4 with acute-on-chronic liver failure, and 2 with primary non-function), MELS used as bridge therapy showed technical viability and safety of the system [89].

Several hurdles with BAL systems need to be surpassed before their effective clinical application, such as the difficulty in reaching the minimum number (45 billion) of hepatic cells required for a clinical-scale BAL, and the high economic cost deriving from the use of large quantities of materials and instruments necessary for cell culture and differentiation, all exponentially increasing depending on treatment length [87].

#### 2.3.3. Bioscaffolds

Through tissue engineering approaches, a number of artificial organs capable of replacing the damaged ones, including heart, bladder, intestines, kidney and liver, have been devised. However, it is not yet possible to recapitulate all the biochemical and architectural complexity of the natural microenvironment to ensure long-term survival and functionality of seeded cells. Decellularized organs may offer a solution [92,93]. Importantly, the protein composition, topography and mechanical properties of the ECM, as well as the microvascular networks for oxygen and nutrient transport, as well as metabolite excretion, in these structures are maintained [92,94].

Although the use of xenogenic livers is promising, the ideal bioscaffold would be decellularised human liver in order to minimize the problems of biocompatibility, immunogenicity and hemodynamics due to the different 3D architecture compared to an animal liver. The first successful decellularization of a human liver and repopulation with derived human liver cells was performed in 2015 by using a novel retrograde, two-step, perfusion flow-rate methodology able to preserve the 3D hepatic architecture and composition, and guarantee excellent viability, motility and cell proliferation [95]. Thereafter, significant progress in the field ensued. It was shown that, under controlled conditions, vascular and biliary networks can also be preserved [96]. Both parenchymal and non-parenchymal cells can be used to repopulate the human liver scaffolds [97]. Moreover, by including human umbilical endothelial cells or HUVEC, these structures can be efficiently revascularised [96,97]. With this breakthrough, some problems related to xenogenic sources of liver for grafts in patients, such as organ size and revascularisation, have been addressed.

Implantable engineered cell-based devices aim at improving metabolic function by providing a small tissue mass (less than 5%), while to restore the liver’s life-saving functions and promote patient survival in case of severe liver diseases, a larger hepatic mass (more than 25%) is required [83]. This, in fact, is one of the major challenges faced to date and has become the main objective of the entire organ decellularisation and recellularisation technology [83]. Even if the full potential of the recellularised human bioscaffolds need to be exploited, one possible use in the clinical setting may be to promote diseased cell replacement following partial hepatectomy (the partial liver scaffolds were sutured onto the surfaces of partially hepatectomised livers) as described in porcine livers [98].

However, there are problems associated with the precise control over the spatial distribution and architectural accuracy of the cells infused in the bioscaffolds. This has been tackled by the introduction of 3D technology bioprinting. This technology allows the development of accurate, detailed and customized engineered structures that mimic tissue and organ functions in vivo and involves indirect and direct manufacturing. The indirect bioprinting initially creates negative sacrificial molds, followed by casting with the desired positive biomaterial and then selective removal of the molds [99]. Instead, the direct ones create 3D structures in a point-by-point and/or layer-by-layer manner, to insert more cell types and/or biomaterials in order to create a structure with reproducibility and heterogeneity as in vivo. The biomaterial used as ink for 3D printing must be biocompatible (to avoid rejection), and with certain viscosity (to determine the correct balance between flexibility and maintenance of structural integrity during and after deposition) [100,101]. The biomaterial based on Pluronic, which is able to pass from the liquid state in solution to the recovery of its shear-thinning hydrogel state at room temperature and upon bioprinting, thus avoiding structural collapse, is an example [102]. The stability of the construct is also determined by the type of crosslinking that can be physical or chemical. The latter proved to be more stable and not subject to dissolution [103]. Liver-like microstructures have been produced with various combinations of hydrogels for hepatocyte production [104]. Interestingly, 3D vascularized liver constructs made of native liver tissue and tight intercellular junctions, with human primary hepatocytes, endothelial cells, and hepatic stellate cells, have proved to be viable and potentially useful for drug screening [105]. The use of these hybrid scaffolds as alternatives to LT needs to be further investigated in clinical studies.

#### 2.3.4. Liver Organoids

Organoids are 3D structures of human tissue that are obtained from primary or stem cells, and are capable of reproducing the architectural and functional properties of diverse cell types present in a full-sized organ (reviewed in [106]). IPSCs, embryonic or adult healthy or diseased tissue-derived stem cells have been employed for organoid formation. Organoids have been used to further hepatic differentiation of stem cells in vitro. For instance, compared to other culture settings, hiPSCs co-cultured with supporting non-parenchymal cells, such as human endothelial cells in 3D spheroids, showed enhanced differentiation and hepatic function in vitro and in vivo [107].

Recently, an unprecedented reproduction of the complex human hepatobiliary pancreatic system was achieved [108]. IPSCs derived from healthy donors successfully generated, over time, interconnected biliary duct and pancreas domains capable of processing bile acids as well as carrying out the pancreatic secretory function (amylase production) in vitro [108]. Hitherto, organoids have provided an excellent tool to study biological processes associated with liver development and regeneration, disease modelling and determination of drug response to offer personalised therapy [109]. By co-differentiating epithelial and stromal lineages derived from human pluripotent stem cells into liver organoids, Ouchi et al. succeeded in modelling the stepwise process leading to steatohepatitis in vitro [110]. Importantly, using atomic force microscopy, changes in stiffness in the fibrotic liver organoids could be monitored efficiently in vitro [110]. Steatohepatitis progressively increases in severity (stepping from liver inflammation and fibrosis to end-stage liver disease) if no therapy is provided. Thus, it is important to identify the right treatment option very early in steatohepatitis-affected patients. Thus, recapitulating precisely a disease in organoids is a significant step forward towards finding patient-specific treatment strategies. Hopefully, in the future, a way of adopting liver organoids in human liver transplant will be found.

## 3. Cell-Free Approach: Extracellular Vesicles

Apart from physically substituting damaged cells in the liver, transplanted cells have paracrine effects (through the secretome) on the microenvironment, thus contributing to the organ regeneration processes (Figure 2). Extracellular vesicles (EVs) are part of the cell’s secretome and are membrane-defined nanoparticles that participate in intercellular and inter-organ communication through exchange of biomolecules (lipids, proteins, and nucleic acid species). Recent advances in the characterisation of EV composition and content have highlighted the importance of EVs for biomarker discovery for different liver pathologies [111].

**Figure 4:**
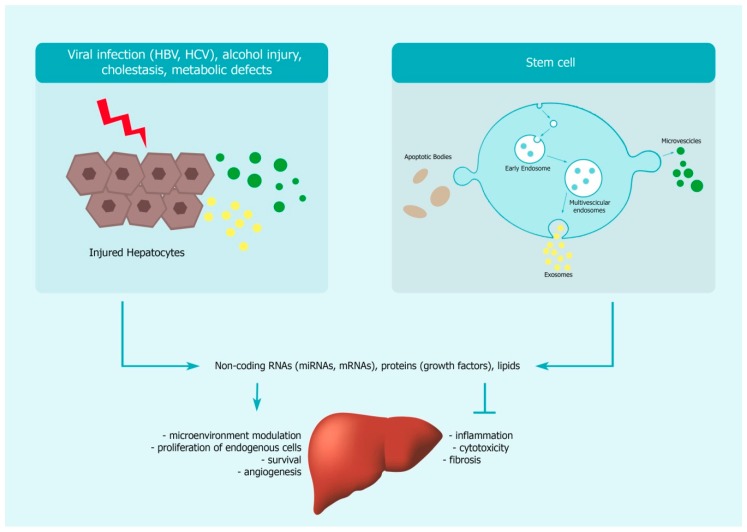
Action of EVs on liver repair. Upon injury, hepatocytes release EVs containing restorative non-coding RNAs, proteins and lipids that induce the regenerative process in the liver by enhancing survival and proliferation of resident cells, neovascularisation, and by modulating niche homeostasis. Stem cell therapy potentiates this process by providing EVs with anti-inflammatory and immunomodulatory properties to the damaged liver. These EVs may have anti-fibrotic effects and prevent cytotoxicity in the liver, hence contributing to slowing the progression to end-stage liver diseases.

EVs also represent a cell-free alternative for the therapy of liver diseases, and may be used as a bridging therapy to LT in some cases (Figure 4). EVs derived from various sources are being assessed for their curative properties in preclinical models of liver diseases. Most studies have hitherto focused on the healing properties of non-coding RNAs (micro-RNAs or miRNAs) present in the EVs [112,113]. Adipose-tissue derived EVs, genetically modified to express miR181-5p for instance, were shown to have anti-fibrotic effects on the liver through autophagy activation and modulation of fibrogenesis-related pathways [114]. In a model of non-alcoholic steatohepatitis, treatment with EVs derived from LSCs (human liver stem cells) significantly alleviated liver inflammation and fibrosis by reprogramming hepatic gene expression through the protein cargo (mainly cytokines and growth factors) contained in the EVs [115]. EVs isolated from iPSCs were also shown to have beneficial effects on the liver by inducing a decrease in expression of profibrogenic markers (α–smooth muscle actin, collagen1α1, fibronectin, and tissue inhibitor of metalloproteinases–1) and responses (chemotaxis and proliferation) in human hepatic stellate cells in vitro, and to reduce liver fibrosis and improve liver function in murine models of liver injury and fibrosis [116]. These effects could be mediated by shuttling of miRNAs harboured by the iPSC-derived EVs (such as miR-92a-3p, miR-26a-5p) into hepatic stellate cells [116]. Long non-coding RNAs present in EVs may also provide beneficial effects. For instance, in the model of fulminant hepatic failure, bone marrow MSC-derived EVs, highly enriched in the long non-coding RNA, Y-RNA-1, dramatically improved survival of mice versus placebo-administered controls by reducing hepatocyte apoptosis [117]. More studies are needed to analyse what happens upon EV treatment in the case of more advanced liver fibrosis or cirrhosis. Moreover, the dosage and frequency of EV administration may be dependent on liver disease type. In preclinical models, such as those of non-alcoholic steatohepatitis and CCL4-induced liver injury, administration of EVs 2 to 3 times per week showed a reduction in profibrotic events in the liver [115,116]. On the other hand, in the bile duct ligation model, daily injection of EVs was required to observe an anti-fibrotic effect [116]. It is also important to assess, in the long-term, the effect of EV (or cell) injection in models with portal hypertension, which develops as a consequence of liver fibrosis, as intravenous delivery may lead to ascites formation (personal observation). All these issues have to be addressed before undertaking human studies. To our knowledge, to date no clinical trials have been undertaken to investigate their therapeutic potential in human liver diseases. This is probably related to the fact that obtaining cost-effective, clinical grade stem cell-derived EVs in sufficient quantity to achieve therapeutic effects in patients has not been attained yet.

## 4. Cell-Based and Cell-Free Gene Therapy for Liver Diseases

With respect to viral vectors that present some limitations for clinical applications, cells offer an alternative platform for gene correction prior to transplantation in the liver [118]. Advantages lie in the fact that gene correction can be efficiently controlled and monitored in vitro, and possible tumorigenic changes assessed, prior to transplantation in patients. Several strategies have been employed to correct genetic defects in stem cells or to reboot genes that modulate liver function. Patient-derived iPSC modelling of liver diseases ex vivo has been used to test the efficiency of exogenous gene delivery or correction. Genome editing strategies, such as TALEN or CRISPR/Cas9 systems have been employed for this purpose [65].

Importantly, as non-viral agents, EVs derived from wild-type human LSCs were also capable of restoring enzymatic deficiency in human LSCs isolated from the liver of a patient with type I citrullinemia, suggesting that these nanometer-sized vesicles can transfer argininosuccinate synthase (ASS1 enzyme) and its mRNA, hence achieving gene therapy for certain inherited disorders [119]. EVs also transfer non-coding RNAs, such as microRNAs (miRNAs) capable of modulating gene expression in the target cells. For instance, MSC-EV-associated miR-122 was successfully transferred to hepatic stellate cells in vitro and inhibited the expression of key genes involved in the synthesis of collagen in these cells [120]. Once identified, the therapeutic miRNAs can be enriched in the EVs, by electroporation into EV or by modulating the expression in the cells of origin, to achieve better efficiency in patients with severe liver diseases [120,121]. It is important to determine which bioactive molecules are harboured by EVs from different cell sources in order to apply patient-tailored therapy in the case of genetic deficiencies. The potential of the cell-free EVs in this direction needs to be fully exploited.

## 5. Conclusions

In an era of organ-shortage crisis, cell-based strategies have made significant leaps forward while keeping pace with the evolving biotechnological advances. However, long-term studies assessing liver histological status post-cell therapy to exclude inflammation and fibrosis as well as biliary problems, in order to ascertain the safety in patients with severe liver diseases, are lacking. The heterogeneity of factors that cause liver failure as well as the patients’ comorbidities can also nuance the benefits of cell-based and cell-free interventions. Moreover, in the case of liver failure, a fully functional, ready-to-use, liver graft is required. All these issues still need to be addressed, and current literature review reveals that, through multidisciplinary efforts, including those of cell and developmental biologists, bio-engineering scientists, immunologists and transplantation surgeons, we are on track for achieving this.

## Figures and Tables

**Table 1:** Some examples of primary hepatocyte transplantation schemes in the clinical setting.

Disease	Donor Type	Conservation Type //Isolation method	Number of cells //Injection route	Outcome	Reference
Urea Cycle disorders	9-day old neonate (post mortem)	Cryopreserved //3-step collagenase perfusion technique	5.6 × 10^9^ //Intraportal	Metabolic stabilisation from 4 to 13 months	Meyburg et al. [34]
Crigler-Najjar Syndrome Type I	5-year old boy (post mortem)	Stored at 4 °C in University of Wisconsin solution //3-step collagenase perfusion	7.5 × 10^9^ //Intraportal	Partial metabolic recovery up to 11 months	Fox et al. [27]
Inherited Factor-VII Deficiency	Unused donor livers	Fresh and cryopreserved //Collagenase perfusion technique	1.09 × 10^9^ 2.18 × 10^9^ //Inferior mesenteric vein	Improvement in coagulation defects; reduced demand for recombinant exogenous factor VII by 20%	Dhawan et al. [35]
Glycogen storage disease type Ia	Unused cadaveric donors	Fresh //2-step collagenase perfusion technique	2 × 10^9^ //Intraportal	Partial correction of metabolic abnormalities (increase in blood-glucose and larger and more persistent inhibition of lactate production compared to before transplantation).	Muraca et al. [28]
Glycogen storage disease type Ib	Unused cadaveric donors	Cryopreserved //2-step collagenase perfusion technique	1st infusion: 1 × 10^9^ 2nd infusion: 3 × 10^9^ //Intraportal	Disappearing of hypoglycemic symptoms;body growth	Lee et al. [36]
Peroxisomal biogenesis disease	Unused left liver segments of two compatible donors	Fresh and cryopreserved //2-step collagenase perfusion technique	2 × 10^9^ //spleno-mesenteric	Improved general condition and weight gain; ability to walk autonomously 6 months after transplantation	Sokal et al. [37]
Acute liver failure by mushroom intoxication	Cadaveric donors	Cryopreserved //Multicatheter collagenase perfusion technique	5 × 10^9^ //4 out of 5 patients: intrasplenic 2 out of 5 patients: intraportal	3 out of 5 patients survived from 12 to 52 days with improvement in clearance function.	Bilir et al. [29]
Argininosuccinate lyase deficiency	Cadaveric donors	Fresh and cryopreserved //2-step collagenase perfusion technique	1st infusion: 7 infusions over 1 month: 1.7 × 10^12^ 2nd infusion: 0.3 × 10^12^ and 0.7 × 10^12^ the day after 3rd infusion: 1 × 10^12^ //Intraportal sequential infusions; portal percutaneous puncture	3.5-year-old patient with sustained metabolic control and clinical evolution of disease from severe to moderate form	Stéphenne et al. [38]

**Table 2:** Clinical trials with LSCs (source: https://clinicaltrials.gov/ and https://www.clinicaltrialsregister.eu/).

NCT Number/EudraCT -Number	Title	Recruitment	Conditions //Intervention	Age //Number of Participants	Phases	Start Date	Outcomes/ Aims
NCT01765243	A Prospective, Open Label, Multicenter, Partially Randomized, Safety Study of One Cycle of Promethera HepaStem in Urea Cycle Disorders (UCD) and Crigler-Najjar Syndrome (CN) Paediatric Patients.	Completed	Urea Cycle Disorders, Crigler Najjar Syndrome //HepaStem infusion	Up to 17 Years //20 participants	Phase I/II	March 2012	Long-term safety profile and preliminary efficacy of HepaStem in paediatric patients with Urea Cycle Disorders and Crigler-Najjar Syndrome
NCT03632148	In Vitro Evaluation of the Effect of HepaStem in the Coagulation Activity in Blood of Patients With Liver Disease	Enrolling by invitation	Decompensated Cirrhosis //Liver MSCs infusion	12 Years to 80 Years //15 participants	N/A	December 2017	Blood parameters in patients with liver disease
NCT03884959	A Prospective, Open Label, Safety and Efficacy Study of Infusions of HepaStem in Urea Cycle Disorders Pediatric Patients	Recruiting	Urea Cycle Disorder //HepaStem infusion	Up to 12 Years //5 participants	Phase II	July 2018	Safety and Efficacy Study of Infusion of HepaStem in Urea Cycle Disorders Pediatric Patients
NCT02946554	Multicenter Phase II Safety and Preliminary Efficacy Study of 2 Dose Regimens of HepaStem in Patients With Acute on Chronic Liver Failure	Recruiting	Acute-on-Chronic-Liver Failure //HepaStem Infusion	18 Years to 70 Years //12 participants	Phase II	December 2016	Safety and Efficacy of 2 Dose Regimens of HepaStem in Patients With Acute on Chronic Liver Failure
NCT03963921	Multicenter, Open-label, Safety and Tolerability Study of Ascending Doses of HepaStem in Patients With Cirrhotic and Pre-cirrhotic Non-alcoholic Steatohepatitis	Recruiting	Nonalcoholic Steatohepatitis //HepaStem infusion	18 Years to 70 Years //24 participants	Phase I/II	April 2019	Evaluation of incidence of Adverse Event
NCT02489292	Prospective, Open Label, Multicenter, Efficacy and Safety Study of Several Infusions of HepaStem in Urea Cycle Disorders Paediatric Patients	Unknown	Urea Cycle Disorders //HepaStem infusion	Up to 12 Years //20 participants	Phase II	October 2014	Efficacy of HepaStem in Urea Cycle Disorders Paediatric Patients
HLSC 01–11, EudraCT-No. 2012–002120-33	Human Liver Stem Cells (HLSCs) in patients suffering from liver-based inborn metabolic diseases causing life-threatening neonatal onset of hyperammonemic encephalopathy	Completed	Inherited Neonatal-Onset Hyperammone-mia	Up to 18 years//3 participants	Phase I	December 2013	Safety and evaluation of short- and long-term clinical and biochemical data after HLSCs injections
